# Encapsulation and 3D culture of human adipose-derived stem cells in an *in-situ* crosslinked hybrid hydrogel composed of PEG-based hyperbranched copolymer and hyaluronic acid

**DOI:** 10.1186/scrt182

**Published:** 2013-03-21

**Authors:** Waqar Hassan, Yixiao Dong, Wenxin Wang

**Affiliations:** 1 Network of Excellence for Functional Biomaterials, National University of Ireland, Galway, IDA Business Park, Dangan, Galway, Ireland

**Keywords:** Human adipose-derived stem cells, Hyaluronic acid, PEG hyperbranched polymers, Hydrogels, Wound healing

## Abstract

**Introduction:**

Cell therapy using adipose-derived stem cells has been reported to improve chronic wounds via differentiation and paracrine effects. One such strategy is to deliver stem cells in hydrogels, which are studied increasingly as cell delivery vehicles for therapeutic healing and inducing tissue regeneration. This study aimed to determine the behaviour of encapsulated adipose-derived stem cells and identify the secretion profile of suitable growth factors for wound healing in a newly developed thermoresponsive PEG–hyaluronic acid (HA) hybrid hydrogel to provide a novel living dressing system.

**Methods:**

In this study, human adipose-derived stem cells (hADSCs) were encapsulated *in situ* in a water-soluble, thermoresponsive hyperbranched PEG-based copolymer (PEGMEMA–MEO_2_MA–PEGDA) with multiple acrylate functional groups in combination with thiolated HA, which was developed via deactivated enhanced atom transfer radical polymerisation of poly(ethylene glycol) methyl ether methacrylate (PEGMEMA, Mn = 475), 2-(2-methoxyethoxy) ethyl methacrylate (MEO_2_MA) and poly(ethylene glycol) diacrylate PEGDA (Mn = 258). hADSCs embedded in the PEGMEMA–MEO_2_MA–PEGDA and HA hybrid hydrogel system (P-SH-HA) were monitored and analysed for their cell viability, cell proliferation and secretion of growth factors (vascular endothelial growth factor, transforming growth factor beta and placental-derived growth factor) and cytokines (IFNγ, IL-2 and IL-10) under three-dimensional culture conditions via the ATP activity assay, alamarBlue^®^ assay, LIVE/DEAD^®^ assay and multiplex ELISA, respectively.

**Results:**

hADSCs were successfully encapsulated *in situ* with high cell viability for up to 7 days in hydrogels. Although cellular proliferation was inhibited, cellular secretion of growth factors such as vascular endothelial growth factor and placental-derived growth factor production increased over 7 days, whereas IL-2 and IFNγ release were unaffected.

**Conclusion:**

This study indicates that hADSCs can be maintained in a P-SH-HA hydrogel, and secrete pro-angiogenic growth factors with low cytotoxicity. With the potential to add more functionality for further structural modifications, this stem cell hydrogel system can be an ideal living dressing system for wound healing applications.

## Introduction

Chronic wound healing has remained difficult despite the advances in tissue engineering such as bioengineered skin substitutes [1]. At least 50% of chronic wounds remain resistant to advanced or standard treatments such as growth factor delivery or bioengineered skin substitutes [2]. In recent years, stem cell-based therapies have emerged as therapeutic alternatives in the regeneration and repair of damaged organs and tissues for various diseases [3]. Recent studies in delayed wound healing animal models using stem cells have been encouraging in treating of chronic wounds [4,5]. Among the many factors contributing to impaired wound healing, the main factors are the reduction of cytokines and reduced neovascularisation [6,7]. Bone marrow-derived stem cells have been shown to modulate the immune response and provide the building blocks for the regeneration of wounds in recent studies [8,9]. Furthermore, adipose-derived stem cells have shown multipotency and secrete many growth factors such as insulin-like growth factor, hepatocyte growth factor, transforming growth factor beta 1 (TGF-β1) and vascular endothelial growth factor (VEGF) that are essential in wound healing and have been shown to heal chronic wounds in preclinical wound healing models [10-13]. In a recent study, human adipose-derived stem cells (hADSCs) embedded in a collagen hydrogel secreted increased levels of growth factors to enhance wound healing [14].

The main strategy to deliver stem cells or growth factors to chronic wounds involves direct injection to the injury site. However, this method leads to poor engraftment and poor efficacy due to proteolytic degradation within the first few hours post injection, thus preventing the beneficial effects of these expensive biological agents [15,16]. These problems can be overcome with the use of a delivery scaffold of natural or synthetic origin. Hydrogel scaffolds have recently been extensively studied because their hydrophilic and injectable properties make them a suitable delivery system for drugs and stem cell therapy [17-21]. Hydrogels can encapsulate stem cells and maintain good viability while keeping their phenotype, which can provide a three-dimensional (3D) microenvironment for stem cells to closely mimic the *in vivo* conditions leading to stimulation of cellular proliferation, differentiation and regeneration via physical or chemical cues [22-24]. However, most of these hydrogel systems are developed via UV crosslinking or require multistep chemically modified reactions and purification methods, which causes safety concerns and increased cost as well as requiring complex preparation methods [25,26].

**Figure 1:**
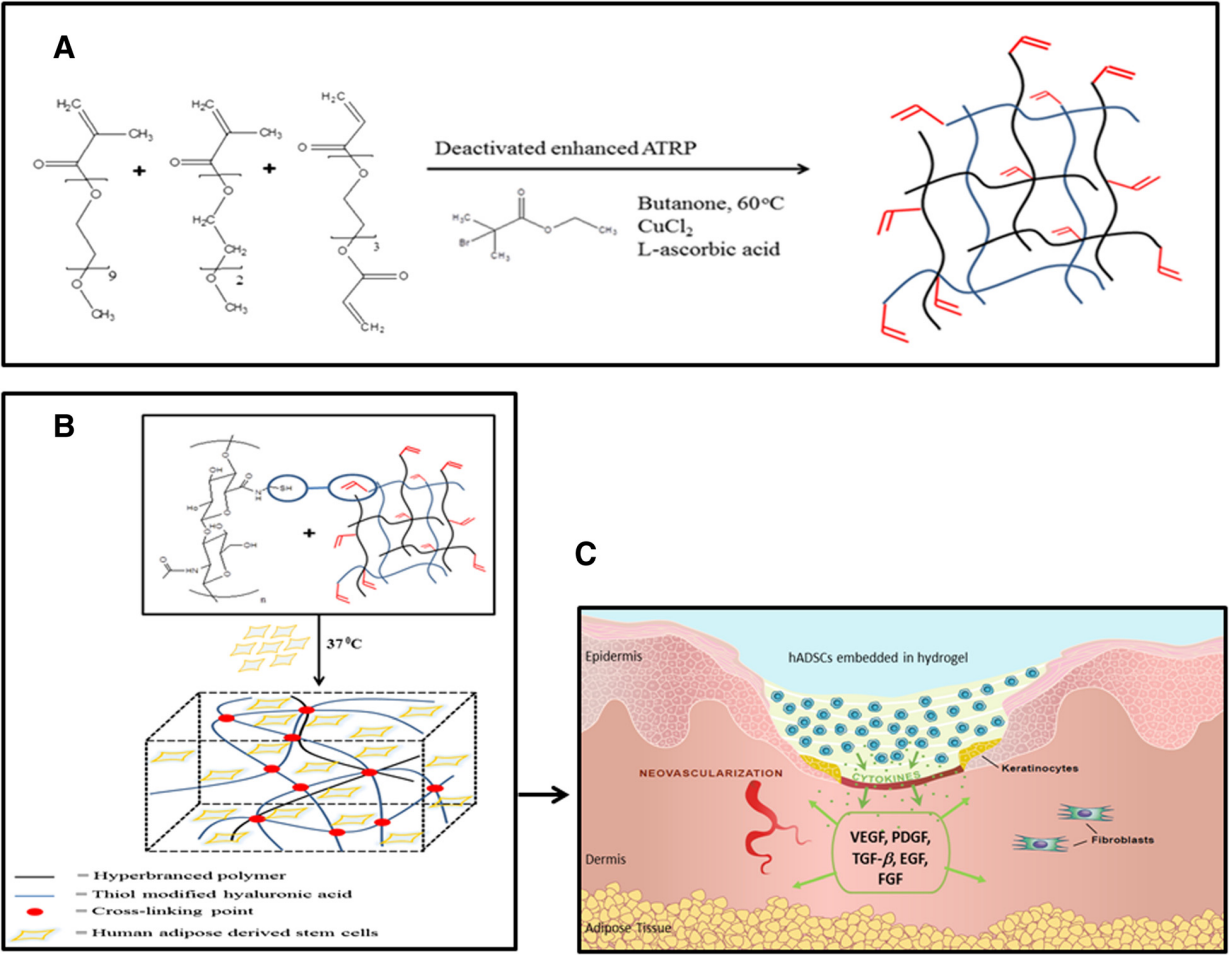
**Schematic illustration of PEGMEMA**_**475**_**–MEO**_**2**_**MA–PEGDA**_**258 **_**copolymer synthesis and cross-linking with thiol-modified hyaluronic acid. (A)** Synthesis route via deactivation-enhanced atom transfer radical polymerisation (ATRP) at 60°C. **(B) **Cross-linking of PEGMEMA–MEO_2_MA–PEGDA and HA hybrid (P-SH-HA) polymer with thiol-modified hyaluronic acid via Michael-type addition reaction showing encapsulation of human adipose-derived stem cells (hADSCs) in the P-SH-HA hydrogel at 37°C. **(C) **Cartoon picture: application of P-SH-HA hydrogel on a skin wound. The hADSCs are embedded *in situ *with P-SH-HA hydrogel and applied on the wound, which secrete growth factors to accelerate wound healing. EGF, epidermal growth factor; FGF, fibroblast growth factor; PDGF, platelet-derived growth factor; TGF-β, transforming growth factor beta; VEGF, vascular endothelial growth factor.

A PEG-based thermoresponsive hyperbranched copolymer of poly(ethylene glycol) methyl ether methacrylate-*co*-2-(2-methoxyethoxy) ethyl methacrylate-*co*-poly(ethylene glycol) diacrylate (PEGMEMA_475_–MEO_2_MA–PEGDA_258_) was developed recently using a one-pot and one-step deactivated enhanced atom transfer radical polymerisation method in our group [27]. This copolymer exhibited a lower critical solution temperature that was close to body temperature. Once the polymer solution was applied at body temperature, a thermal gelation rapidly occurred. In addition, with high content of vinyl functional groups, this polymer further crosslinks chemically with thiolated hyaluronan (HA-SH) in minutes via a Michael-type thiolene reaction, which increases the mechanical properties of the hydrogel. This thermoresponsive hydrogel system allows *in situ* encapsulation of stem cells in a very short period of time, which can be used to deliver cells and growth factors. The secretion of growth factors from embedded cells could help induce the healing process in chronic wounds (Figure 1C).

The purpose of this study is to analyse this system for soft-tissue engineering by successful encapsulation of hADSCs *in situ*. The effects of the PEGMEMA–MEO_2_MA–PEGDA and HA hybrid (P-SH-HA) hydrogel on cellular toxicity, proliferation and secretion of proinflammatory/anti-inflammatory and angiogenic growth factors were examined. Herein, we describe the preliminary characterisation of P-SH-HA synthetic extracellular matrix hydrogels for use in cell culture *in vitro* and with potential application *in vivo*.

## Methods

### Materials

The chemicals PEGMEMA (Mn = 475 g/mol), MEO_2_MA, and PEGDA (Mn = 258 g/mol) were purchased from Sigma-Aldrich (Wicklow, Ireland). HA-SH (HyStem™, Glycosan) was purchased from BioTime Inc. (Alameda, CA, USA). All chemicals were of analytical grade.

### PEGMEMA–MEO_2_MA–PEGDA polymer synthesis and characterisation

The PEGMEMA–MEO_2_MA–PEGDA copolymer was synthesised by the copolymerising of PEGMEMA, MEO_2_MA and PEGDA via an *in situ* deactivation-enhanced atom transfer radical polymerisation approach as previously described [28]. Briefly, PEGMEMA (7.4 g, 0.015 moles), MEO_2_MA (12.8 g, 0.068 moles), PEGDA (5.4 g, 0.021 moles), the initiator ethyl 2-bromoisobutyrate (155 μl, 0.001 moles), copper(II) chloride (0.032 g, 0.0002 moles), bis(2-dimethylaminoethyl)methylamine (64 μl, 0.0002 moles) were added to a two-neck flask in 25 ml solvent butanone. The mixture was stirred for complete dissolution followed by purging with argon for 30 minutes to remove dissolved oxygen. l-Ascorbic acid (0.011 g) was added to the polymerisation solution under argon conditions and the mixture was heated in an oil bath to 50°C and stirred for 6 hours.

The polymerisation was stopped by opening the flask and exposing the catalyst to air. After the polymerisation, the solution was diluted with (1:1) acetone and precipitated into a large excess of diethyl ether and hexane (1:1.2) to remove solvent and monomers. The precipitated mixture of the polymer was dissolved in deionised water and purified by dialysis (spectrum dialysis membrane, molecular weight cutoff 6,000 to 8,000) for 72 hours in a dark environment at 4°C against fresh deionised water, while the water was changed regularly. The pure polymer samples were obtained after freeze drying. The molecular weight and molecular weight distributions were determined for PEGMEMA–MEO_2_MA–PEGDA using gel permeation chromatography (Polymer Laboratories) (Amherst, MA, USA) with an (Refractive Index) detector using dimethylformamide as an eluent. The columns (30 cm PLgel Mixed-C, two in series) were calibrated with poly (methyl methacrylate) standards. All calibrations and analysis were performed at 60°C and a flow rate of 1 ml/minute. ^1^H-NMR was carried out for PEGMEMA–MEO_2_MA–PEGDA on a 300 MHz Bruker NMR with MestReC processing software. The chemical shifts were referenced to the lock chloroform (CDCl_3_) for PEGMEMA–MEO_2_MA–PEGDA (Sigma, Wicklow, Ireland).

### Hydrogel preparation

Commercially available HA-SH (Glycosan-HyStem™; BioTime Inc.) was used for making hydrogels,. The hydrogel was prepared by reacting the vinyl groups on the hyperbranched copolymer with free thiol contents of HA-SH at physiological condition via Michael-type addition. For this purpose, HA-SH (1% w/v) and PEGMEMA–MEO_2_MA–PEGDA (10% wt) were prepared in PBS (pH 7.4) in separate glass vials. The two solutions were combined in a volume ratio of 1:1 and gently mixed for 30 seconds. The solution was than incubated at 37°C to obtain gels for further analysis.

### Cell culture

STEMPRO^®^ hADSCs (Invitrogen) (Life Technologies, Dublin, Ireland) isolated from human adipose tissue collected during liposuction procedures were obtained at passage 1. The cells were grown and passaged using MesenPRO RS™ Medium (includes basal medium and growth supplement; Invitrogen) during all of the studies and used between passages 2 and 4. These cells have been shown to express a cell-surface protein profile that is positive for CD29, CD44, CD73, CD90, CD105, and CD166 (>95%), and is negative for CD14, CD31, CD45, and Lin1 (<2%).

### Two-dimensional and three-dimensional cell culture

For two-dimensional (2D) culture, hADSCs were seeded on a 96-well plate (2D) at a final concentration of 0.5×10^6^ cells/ml. Similarly, for 3D culture the cell suspension was mixed with PEGMEMA–MEO_2_MA–PEGDA and HA-SH at a final concentration of 0.5×10^6^ cells/ml. After gelation, the cell laden gel constructs were transferred to a 48-well plate and 0.5 ml MesenPRO RS™ media were added supplemented with 2% growth supplement (Invitrogen). The media was changed every second day. Tissue culture plastic (TCP) seeded cells were used as experimental controls in all experiments.

### Cell viability

The alamarBlue^®^ (Invitrogen) assay was performed to evaluate the metabolic activity of hADSCs in the 3D cell culture system. hADSCs were encapsulated in 3D hydrogels as described above. At each time point, cells were washed three times with PBS at 37°C, following which 10% alamarBlue^®^ in MesenPRO RS™ cell culture media was added to the wells containing hydrogel constructs to assess the cell metabolic activity after 1, 3, 5 and 7 days. Viability of encapsulated cells was also measured via intracellular ATP activity using Celltiter-Glo^®^ reagent (Promega) (Madison, WI, USA) at days 2, 5 and 7. Media from cell-laden hydrogels were removed and replaced with 200 μl of 50% Celltiter-Glo^®^ reagent in MesenPRO RS™ media. Following incubation on a shaker for 45 minutes at room temperature, 150 μl of the solutions were transferred into a 96-well white plate for luminescence quantification using a microplate reader. The LIVE/DEAD^®^ assay (Molecular Probes) (Life Technologies, Dublin, Ireland) was also used to visualise the distribution of living and dead cells in the hydrogel at different time points for the 3D culture system. Fluorescence images were taken using an Olympus Fluoview Confocal Microscope.

### Cell proliferation assay

Cell proliferation was determined using PicoGreen^®^ fluorescent DNA quantification (Molecular Probes) kit at days 2 and 7 for both 3D and 2D culture as described by the manufacturer. DNA from cell-laden hydrogels was recovered by first mechanically digesting hydrogels and then enzymatically in proteinase K overnight at 56°C. Briefly, 100 μl DNA samples were incubated with 100 μl diluted (1:200) PicoGreen^®^ reagent in 1× TE buffer in a 96-well opaque, flat-bottomed assay plate. The fluorescence was read at excitation 485 nm and emission 525 nm and was compared with a DNA standard curve provided with the kit.

### Determination of growth factor secretion

After each predetermined time point (days 1, 3, 5 and 7), conditioned media from both 2D and 3D culture systems was collected and stored at -80°C for later analysis of the secreted factors using a multiplex ELISA system. The media was not changed in these samples until 7 days. The cell-laden hydrogels (3D) and cells on TCP (2D) were also harvested and stored at -80°C until further analysis. The conditioned media of hADSCs were analysed (days 1, 3, 5 and 7) for both the angiogenic growth factors VEGF, placental-derived growth factor (PlGF), TGF-β and the proinflammatory/anti-inflammatory cytokines IFNγ, IL-2 and IL-10 by the Mesoscale development system with multiplex ELISA kits (MSD TH1/TH2 7-plex, MSD Human Growth factor 1 4-Plex) (Meso Scale Discovery, Gaithersburg, MD, USA) as described by the manufacturer.

### Statistical analysis

All data are expressed as mean ± standard deviation of triplicate samples. Comparisons between multiple groups were performed using one-way ANOVA. All analyses were performed with GraphPad Prism 5 (CA, USA). Differences between two datasets were considered significant when *P* <0.05.

## Results and discussion

3D culture systems have frequently been used to mimic *in vivo* conditions. These systems allow for the control of cellular behaviour by providing control over properties of material substrates and microenvironments using natural or synthetic materials. This control allows creation of a stem cell niche to maintain or control the stemness of cells. Many synthetic and biological 3D systems such as collagen, hyaluronic acid, PEG-based synthetic extracellular matrix (QGEL™ ) (Lausanne, Switzerland) and chitosan have been reported recently [24]. However, most of these systems are synthesised via chemical modifications, which require multistep reactions and purification methods leading to increased production costs as well as giving rise to complex preparation methods. To overcome these problems, a hyperbranched PEG-based copolymer was therefore recently synthesised using a one-pot and one-step synthesis method (Figure 1A). This synthesis creates a hydrogel with thermoresponsive properties and desired end functionality to provide an *in situ* crosslinking system that can crosslink with HA-SH via Michael-type addition (Figure 1B). This hydrogel system allows encapsulation of stem cells *in situ*, which could potentially be used as a system to form a dressing upon application for wound repairs (Figure 1C). Understanding stem cell behaviour and analysing the ability for cells to secrete growth factors is therefore essential. In this paper, PEG-based thermoresponsive copolymer cross-linked with HA-SH was studied for hADSC viability, proliferation and secretion of pro-angiogenic growth factors and inflammatory cytokines under 3D culture condition. Cells seeded on TCP (2D) were used throughout the experiments to provide experimental control for the secretion of growth factors, proliferation of cells and viability.

### PEGMEMA–MEO_2_MA–PEGDA polymer synthesis and characterisation

Synthesis and characterisation of the PEGMEMA–MEO_2_MA–PEGDA copolymer was carried out as previously reported [28]. The reaction for the polymer was stopped when the desired molecular weight (70 kDa) was reached. The hyperbranched structure of the polymer was confirmed by ^1^H-NMR and peaks at chemical shift of 6.4 to 5.8 ppm proved the presence of a vinyl functional group as described previously [28].

### Cell morphology in P-SH-HA hydrogel

**Figure 2:**
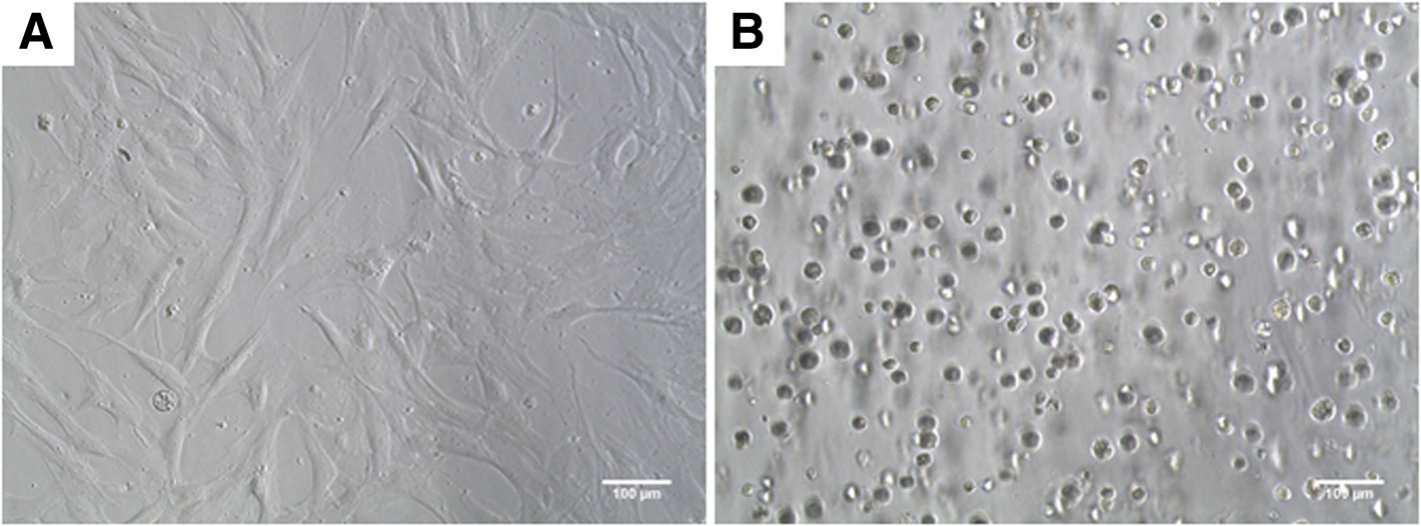
**Phase images of tissue culture plastic and hydrogel encapsulated human adipose-derived stem cells. **Phase image of tissue culture plastic (TCP; two-dimensional) and hydrogel (three-dimensional) encapsulated human adipose-derived stem cells (hADSCs) exhibiting **(A) **fibroblastic-like morphology and **(B)** rounded morphology after 2 days of seeding. hADSCs were seeded at a final concentration of 0.5×10^6 ^cells/ml in a 96-well plate.

Cellular morphology is an important phenomenon that is correlated with important biochemical functions involved in new tissue formation such as proliferation and migration of cells. It is known that cells encapsulated in PEG-based hydrogels without any attachment sites adopt rounded morphology unless some extracellular matrix materials are present [29]. The cell morphology for hADSCs was therefore evaluated within the hydrogels (3D) after encapsulation. It was noted the hADSCs were stretched with spindle-like shape on 2D culture as expected. In contrast, the cells in hydrogels lost this morphology and showed more rounded, isolated distribution throughout the gel (Figure 2). The spreading, migration and attachment of cells in the 3D microenvironment is dependent on many different factors; mainly substrate density, stiffness, elastic modulus, degradation and adhesion within the microenvironment of the hydrogels. Cells seeded on HA hydrogels have been shown to spread via CD44 and its co-receptor RHAMM [30]. In other studies, however, encapsulated cells in HA hydrogels do not spread but maintain good viability [30,31]. Our results agree with the latter studies, suggesting that hADSCs can remain viable in P-SH-HA copolymer without spreading. This could be due to the lack of presentation of attachment sites and receptors because of the highly cross-linked network.

### Cellular viability in P-SH-HA hydrogel

**Figure 3:**
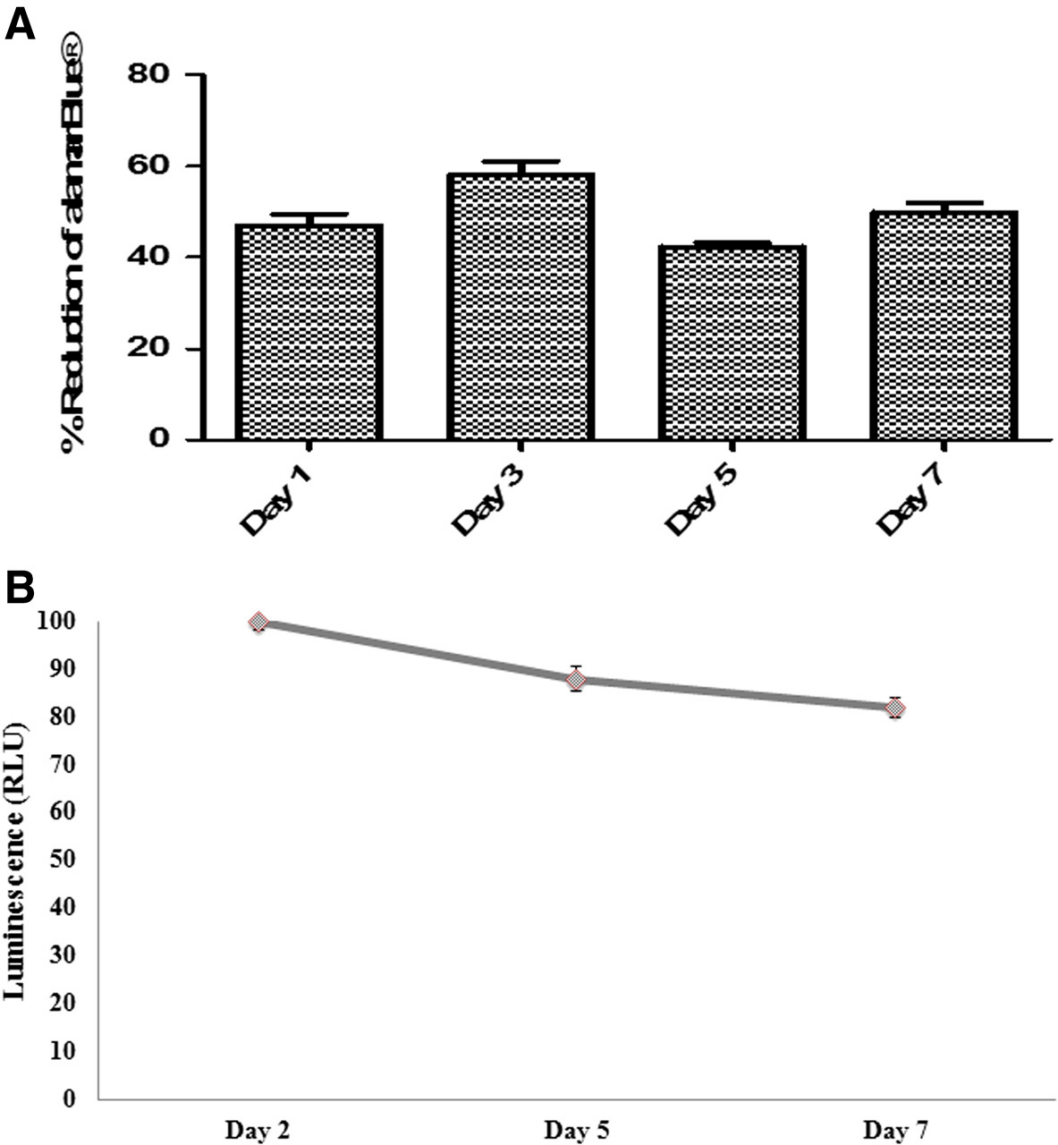
**Cellular metabolic activity and viability of encapsulated human adipose-derived stem cells. (A) **The alamarBlue^®^ metabolic assay (Invitrogen ) (Life Technologies, Dublin, Ireland) for encapsulated human adipose-derived stem cells (hADSCs) in PEGMEMA–MEO_2_MA–PEGDA and HA hybrid hydrogels at days 2, 5 and 7 (mean ± standard deviation, *n = *3). **(B) **Intracellular activity of ATP of hADSCs was measured at the indicated time after encapsulation and was used to represent cell metabolic activity and viability (mean ± standard deviation, *n = *3). RLU, relative luminescence units.

To evaluate the biocompatibility of the hydrogels with hADSCs, quantitative cellular metabolic activity and viability were assessed using the alamarBlue^®^ assay and the ATP assay, respectively. Qualitative analysis was carried out via the LIVE/DEAD^®^ assay. hADSCs were encapsulated in hydrogel scaffold at a final concentration of 0.5×10^6^ cells/ml for up to 7 days. Unseeded hydrogels alone were used as negative control and tissue culture plastic (2D) seeded hADSCs were used as positive experimental controls. The alamarBlue^®^ assay was utilised to test the viability of encapsulated cells at each time point. It was noted that after day 7 (49%), cellular activity remained stable in comparison with day 1 (47%). This indicates that there was no cytotoxic effect on cell metabolic activity from the hydrogel system (Figure 3A). Similar results were shown by ATP intracellular activity, which remained above 82% for 7 days (Figure 3B).

**Figure 4:**
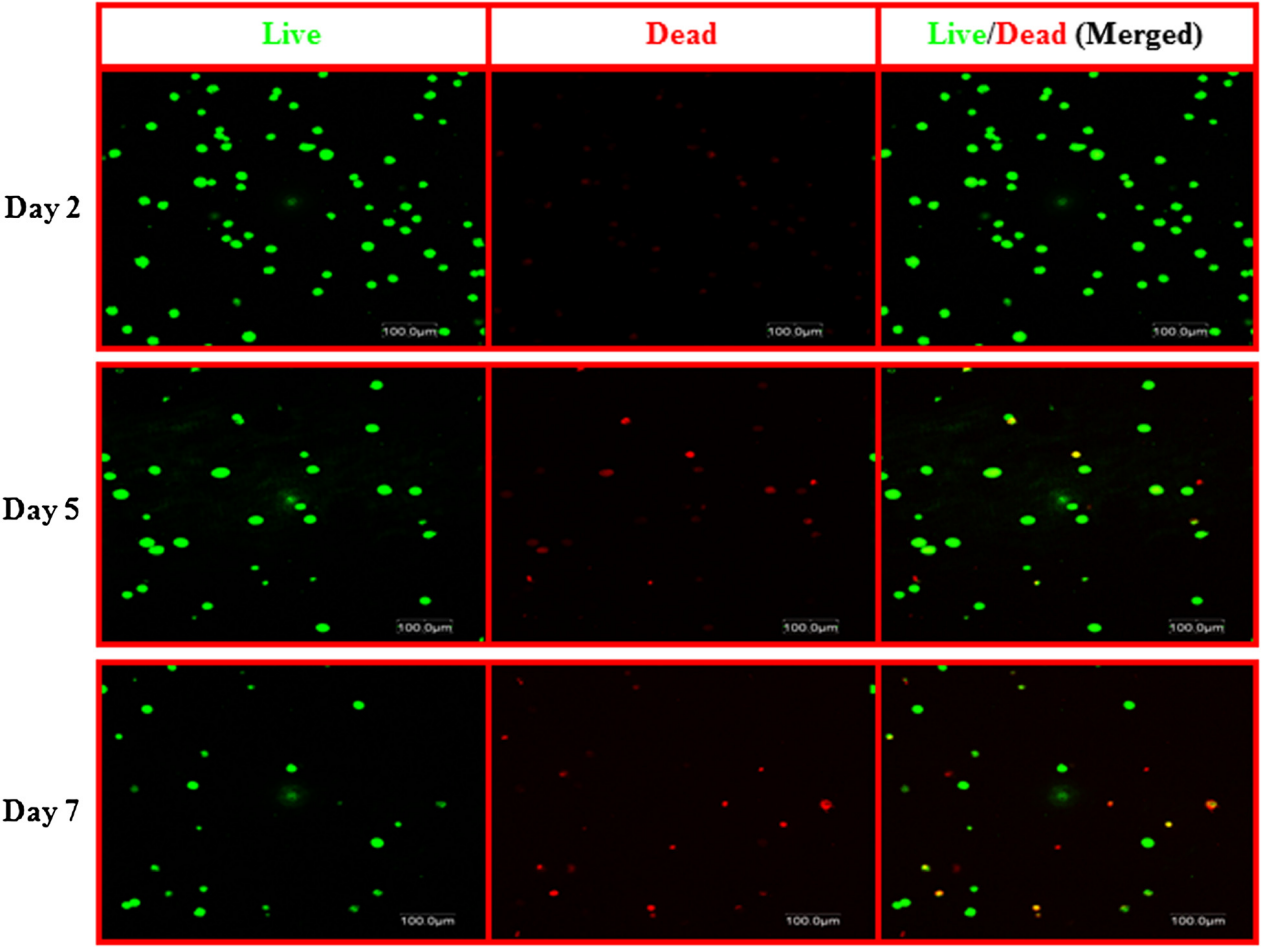
**Cellular viability with the LIVE/DEAD^®^ cell viability assay. **Representative confocal Z-stack (300 μm) images of encapsulated human adipose-derived stem cells stained with calcein AM (live) and ethidum homodimer (dead) viability staining kit at days 2, 5 and 7 using the LIVE/DEAD^®^ assay (Molecular Probes) (Life Technologies, Dublin, Ireland). Live cells were stained green while dead cells were stained red. Scale bar: 100 μm.

The LIVE/DEAD^®^ assay (Molecular Probes) was employed to visualise the distribution of living and dead cells after 2, 5 and 7 days in the 3D cell culture system (Figure 4). Calcein AM fluoresces green upon the reaction of intracellular esterase and stains live cells; ethidium homodimer-1, which binds to the DNA of dead membrane compromised cells, stains dead cells (red). Few dead cells appeared after 5 days; however, the number of live cells was obviously much higher even after 7 days. We assumed that the hydrogel density that was increased further by thermal gelation in addition to chemical gelation and the nondegradable property of the hydrogel have limited the living space for the encapsulated cells and that the exchange of nutrients may not have been efficient and could have caused a slight drop in cell viability after 7 days. Overall, one can conclude that this nontoxic *in situ* gelling system can provide a suitable microenvironment in which hADSCs remain viable for up to 7 days.

### Cellular proliferation in P-SH-HA hydrogel

**Figure 5:**
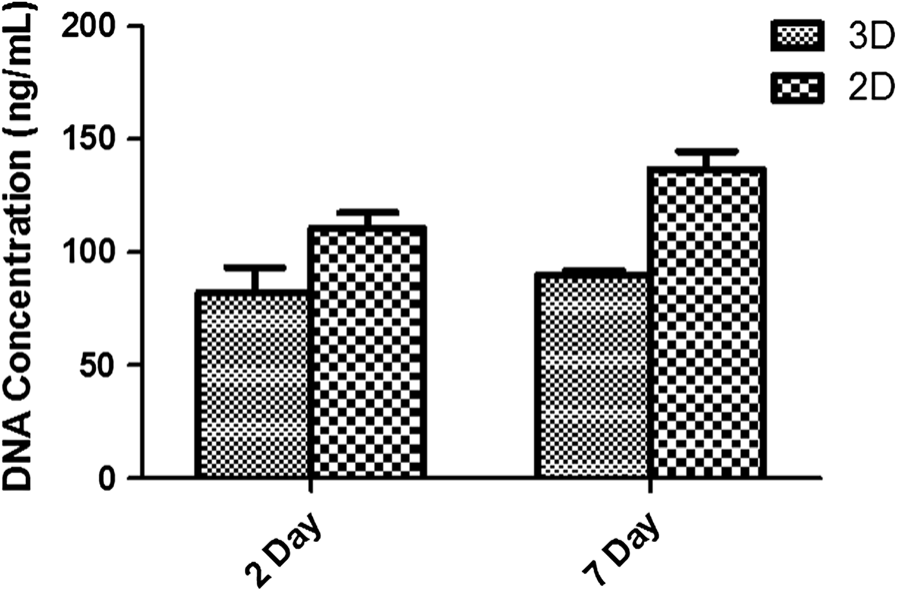
**Proliferation rates of human adipose-derived stem cells in three-dimensional and two-dimensional cell culture conditions. **Cells were seeded at 0.5×10^6^/ml. Cellular proliferation was determined by PicoGreen^®^ assay (Molecular Probes) (Life Technologies, Dublin, Ireland) after recovering the DNA from cells in both three-dimensional (3D) and two-dimensional (2D) culture systems. There was no significant difference in cell proliferation rates in both 3D and 2D cultures (*n* = 3, *P *<0.05).

The cell-encapsulated hydrogel samples prepared as mentioned above were digested mechanically and enzymatically with proteinase K prior to analysis. The DNA content was recovered and analysed with PicoGreen^®^ assay after day 2 and day 7. The concentration of DNA from encapsulated cells was maintained at similar levels across both time periods; that is, 82.12 ± 19.20 ng/ml at day 2 and 90.15 ± 3.80 ng/ml at day 7 (Figure 5). The proliferation rate from 3D seeded cells was significantly lower than that for 2D seeded cells. This phenomenon has been reported elsewhere and might be attributable to the cellular microenvironment [32]. These results indicate that the hADSCs did not proliferate in hydrogel even after 7 days. For spreading and migration of cell in a 3D structure, they need to be able to degrade the polymeric structure or extracellular matrix. HA is a degradable molecule; however, when cross-linked with a PEG-based polymer it can become difficult for cells to degrade. Cells secreting hyaluronidase can degrade the P-SH-HA scaffold partially but this degradation is not sufficient to create enough space for cells to move around and proliferate. Our results here agree with other reports suggesting that nondegradable HA hydrogels suppress proliferation of mammalian cells, but can maintain viability [32].

### Secretion analysis of angiogenic and inflammatory cytokines

Growth factors and cytokines are important mediators that allow cell–cell communication between other cell types that have been shown to play important roles in normal physiological and pathological processes. For example, proinflammatory cytokines are mainly involved in early stages of wound healing via inflammatory cells and if not properly regulated can lead to abnormal wound healing. In response to different materials, cells can react and produce inflammatory cytokines. Analysing the cellular response to newly developed biomaterials is therefore necessary. For this purpose we selected IL-2 and IFNγ, which are proinflammatory cytokines and may lead to detrimental effects if not controlled properly.

**Figure 6:**
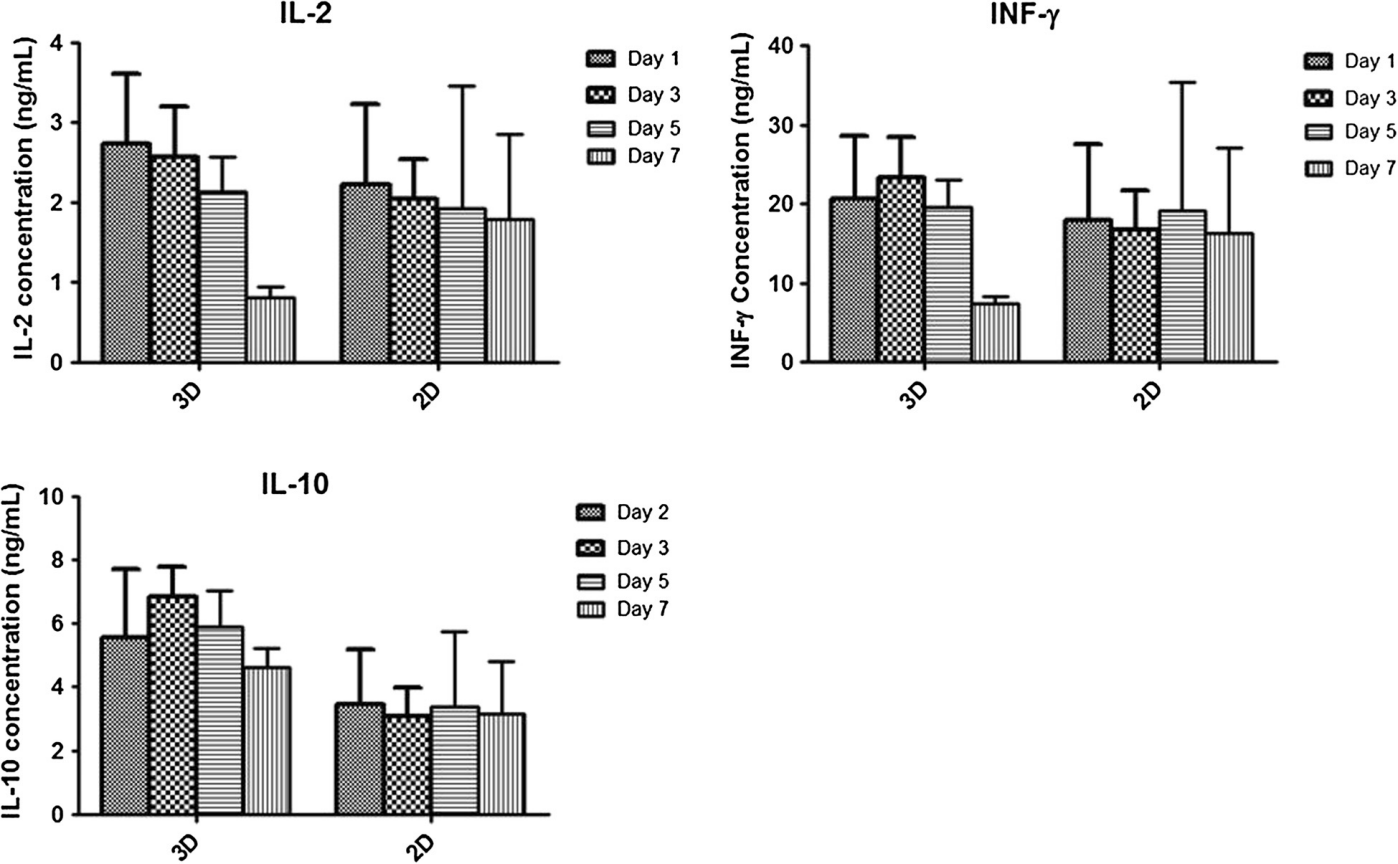
**Secretion profile for cytokines of human adipose-derived stem cells in three-dimensional and two-dimensional cell culture. **Secretion profile of cytokines of human adipose-derived stem cells in both PEGMEMA–MEO_2_MA–PEGDA and HA hybrid hydrogel (three-dimensional (3D)) and tissue culture plastic (two-dimensional (2D)) over 7 days. Cells were seeded at 0.5×10^6^ cells/ml in both 3D and 2D culture conditions in 48-well plates. The supernatant from both conditions was analysed using a multiplex ELISA kit for IL-2, IL-10 and IFNγ (*n *= 3, *P *<0.05).

From our results, the inflammatory cytokines analysed were interestingly reduced from 28.38 ± 5.14 ng/ml at day 1 to 7.38 ± 1.60 at day 7 ng/ml for IFNγ (Figure 6) and from 2.73 ± 1.52 ng/ml at day 1 to 0.82 ± 0.21 ng/ml at day 7 for IL-2 in the 3D microenvironment (Figure 6). Media from cells seeded on TCP were used to analyse the 2D and 3D conditions for all time-points. TCP seeded cells acted as positive controls to measure cytokines and growth factors for ELISA because hADSCs have shown to secrete a detectable amount of growth factor in 2D conditions. There was no significant difference in secretion of IFNγ and IL-2 at any time point for TCP seeded cells. This indicated that the P-SH-HA hydrogel scaffold did not induce an inflammatory response and that the hydrogel is a suitable scaffold system for cell encapsulation. For IL-10, an anti-inflammatory cytokine that has been reported to be beneficial in dampening down the immune response and can aid in impaired wound healing, no significant difference was observed in both 3D and 2D culture. However, it was noted that levels of IL-10 started to drop after day 3 in 2D culture but remained at stable levels in 3D culture over 7 days (4.60 ± 1.07 ng/ml), which may provide an indication that presence of HA in the hydrogel scaffold can modulate an anti-inflammatory response from the encapsulated cells. The secretion analysis suggests that the hydrogel scaffold developed here does not induce an inflammatory reaction from the encapsulated cells and that maintaining levels of IL-10 an anti-inflammatory cytokine may be beneficial for applications in wound healing.

Hydrogel systems have been recently used to deliver growth factors such as VEGF, keratinocyte growth factor, basic fibroblast fibroblast growth factor and hepatocyte growth factor for enhancing the wound healing process [33-35]. This delivery does not translate well in clinical settings, however, as is the case with becaplermin that consists of platelet-derived growth factor [36]; administering single growth factor to enhance angiogenesis in atherosclerotic patients has also shown little success [37]. During wound healing, multiple growth factors are known to work in synergistic fashion to provide a positive outcome [38]. The secretion of anti-apoptotic and angiogenic growth factors from stem cells can therefore provide a valuable tool for angiogenic therapies. In recent studies, hADSCs have been shown to enhance wound healing effectively by both directly differentiating or providing necessary growth factors and cytokines at local sites by enhancing angiogenic response [10]. These beneficial effects of hADSCs have been observed in ischemic models via promotion of angiogenesis by secretion of angiogenic cytokines such as VEGF and hepatocyte growth factor [39,40]. Therefore, after analysing the inflammatory cytokines, we decided to test for the secretion of pro-angiogenic growth factors.

**Figure 7:**
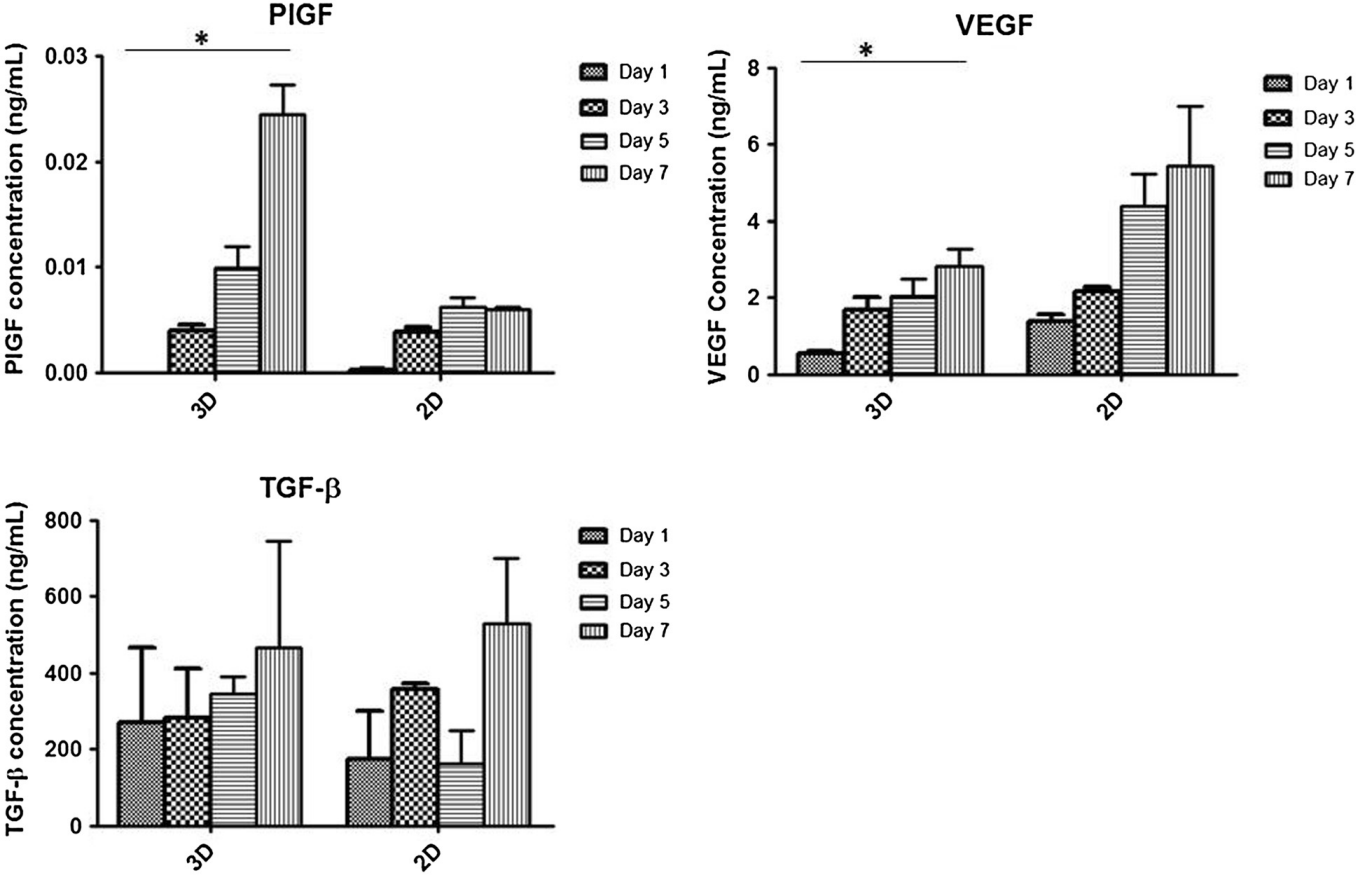
**Secretion profile for growth factors of human adipose-derived stem cells in three-dimensional and two-dimensional culture. **Secretion profile of growth factors of human adipose-derived stem cells in both PEGMEMA–MEO_2_MA–PEGDA and HA hybrid hydrogel (three-dimensional (3D)) and tissue culture plastic (two-dimensional (2D)) over 7 days. Cells were seeded at 0.5×10^6^ cells/ml in both 3D and 2D culture conditions in 48-well plates. Supernatant from both conditions was analysed using a multiplex ELISA kit for placental-derived growth factor (PlGF), vascular endothelial growth factor (VEGF) and transforming growth factor beta (TGF-β) (*n *=3, *P *<0.05). *Statistical significance.

For this purpose, we chose PlGF, VEGF and TGF-β. PlGF is a member of the VEGF family and both of these growth factors are potent angiogenic factors along with TGF-β, which is present in the initial stages of wound healing and regulates inflammatory cellular responses. hADSCs were seeded in P-SH-HA hydrogels at a density of 0.5×10^6^ cells/ml. Supernatant was collected at days 1, 3, 5 and 7 after seeding. Cells plated on TCP were also used to analyse secretion. From the results it was noted that production of PlGF increased in both conditions (2D and 3D) over 7 days; however, there was significantly more PlGF present in the 3D culture system (0 ng/ml at day 1 to 0.025 ± 0.01 ng/ml at day 7) at 7 days when compared with day 1 (Figure 7). We also noted that VEGF production is lower in 3D culture than in 2D culture over all time points but the production of VEGF increased (0.53 ± 0.05 ng/ml) over time from day 1 to day 7 (2.81 ± 0.25 ng/ml); a similar trend was noted in the media from TCP seeded cells (Figure 7). TGF-β was also tested for release and showed increased production over time in both conditions (2D and 3D), but there was no significant difference in concentration levels at any time for 3D culture. These results suggest that hADSCs can secrete growth factors, albeit at low levels. PlGF interestingly increased over 7 days; however, the levels secreted were very low. It is important to note that chemical and mechanical properties of hydrogel can modulate the stem cell response; therefore it is necessary to test different conditions to optimise the production of pro-angiogenic growth factors. In conclusion, it can be observed that secretion and maintenance of pro-angiogenic and anti-inflammatory cytokines is possible in this P-SH-HA hydrogel system. The next step is therefore optimising this hydrogel system to increase the levels of secreted growth factors.

## Conclusion

This study shows that hADSCs can be successfully encapsulated in a newly formed *in situ* P-SH-HA hydrogel system to support the maintenance of hADSCs and to secrete essential growth factors. P-SH-HA hydrogel does not induce cytotoxicity over a 7-day period as measured by cell viability with live/dead staining and the alamarBlue^®^ assay. hADSCs successfully secreted pro-angiogenic growth factors (PlGF, VEGF and TGF-β) and did not induce toxicity or inflammatory response as evident by low production of proinflammatory cytokines (IFNγ and IL-2). This *in situ* thermoresponsive copolymer with multiple acrylate groups has many advantages for the potential applications in tissue engineering and wound healing applications. The thermoresponsive property of this hydrogel allows easy handling during clinical practice and application as a dressing system. Furthermore, the multiple acrylate groups within the copolymer can provide tuneable mechanical properties leading to different swelling properties, to release profiles of growth factors from the gels and to allow control over secretion and cellular processes. Taken together, these results indicate that the PEG-HA-based synthetic extracellular matrix hydrogel can be used for topical application to deliver cells for wound healing applications.

## Abbreviations

2D: Two-dimensional; 3D: Three-dimensional; ELISA: Enzyme-linked immunosorbent assay; HA: Hyaluronic acid; HA-SH: Thiol-modified hyaluronic acid; hADSC: Human adipose-derived stem cell; IFN: Interferon; IL: Interleukin; MEO2MA: 2-(2-methoxyethoxy)ethyl methacrylate; NMR: Nuclear magnetic resonance; P-SH-HA: PEGMEMA–MEO_2_MA–PEGDA and HA hybrid; PBS: Phosphate-buffered saline; PEG: Polyethylene glycol; PEGDA: Poly(ethylene glycol) Diacrylate; PEGMEMA: Poly(ethylene glycol) methyl ether methylacrylate; PlGF: Placental-derived growth factor; RHAMM: Receptor for hyaluronan-mediated motility; TCP: Tissue culture plastic; TGF-β: Transforming growth factor beta; VEGF: Vascular endothelial growth factor.

## Competing interests

The authors declare that they have no competing interests.

## Authors’ contributions

WW conceived the idea and designed the experiments, and WH and YD executed all of the experiments. WH, YD and WW interpreted and analysed the data and drafted the manuscript. WH was responsible for cell culture experiments, collection and assembly of data. YD contributed to the preparation of hydrogel materials. All authors read and approved the final manuscript.
